# A comparative analyses of lipid ratios representing desaturase enzyme activity between preterm and term infants within the first ten weeks of life

**DOI:** 10.1186/s12944-023-01862-8

**Published:** 2023-08-23

**Authors:** Hanis Hidayu Kasim, Laurentya Olga, Stuart Snowden, Eliza Cropp, Albert Koulman, Kathryn Beardsall

**Affiliations:** 1 Department of Paediatrics, University of Cambridge, Cambridge Biomedical Campus, Hills Road, Cambridge, CB2 0QQ UK; 2 Wellcome-MRC Institute of Metabolic Science-Metabolic Research Laboratories, University of Cambridge, Cambridge, UK; 3 Neonatal Unit, University of Cambridge Addenbrookes Hospitals NHS Foundation Trust, Cambridge, UK

**Keywords:** Desaturase enzyme, Preterm infants, LCPUFAs, Metabolism

## Abstract

**Background:**

Desaturase enzymes play a key role in several pathways including biosynthesis of poly- and mono- unsaturated fatty acids (PUFAs, MUFA). In preterm infants, desaturase enzyme activity (DA) may be a rate-limiting step in maintaining PUFAs levels during this critical developmental window and impact on long term metabolic health. The study tested the hypothesis that DA is altered in preterm infants compared to term infants in early life and may be a marker of risk or contribute to later alterations in metabolic health.

**Methods:**

Lipidomic analyses were conducted using blood samples from two established UK-based cohorts, involving very preterm (n = 105) and term (n = 259) infants. Blood samples were taken from term infants at birth, two and six weeks and from preterm infants when established on enteral feeds and at term corrected age. DA of the 2 groups of infants were estimated indirectly from product/precursor lipids ratios of phosphatidylcholine (PC) and triglycerides (TG) species and reported according to their postmenstrual and postnatal ages.

**Results:**

There were changes in lipid ratios representing desaturase enzyme activity in preterm infants in the first weeks of life with higher delta 6 desaturases (D6D) triglyceride (TG) indices but significantly lower delta 9 desaturase (D9D) and D6D(PC) indices. In comparison to term infants, preterm have lower delta 5 desaturase (D5D) but higher D6D indices at all postnatal ages. Although point levels of desaturase indices were different, trajectories of changes in these indices over time were similar in preterm and term infants.

**Conclusions:**

This study findings suggest the patterns of desaturase indices in preterm infants differ from that of term infants but their trajectories of change in the first 10 weeks of life were similar. These differences of DA if they persist in later life could contribute to the mechanism of diseases in preterm adulthood and warrant further investigations.

**Supplementary Information:**

The online version contains supplementary material available at 10.1186/s12944-023-01862-8.

## Background

Desaturase enzymes play a key role in a number of biochemical pathways including biosynthesis of poly- and mono- unsaturated fatty acids (PUFAs, MUFAs). Altered desaturase activity has been associated with increased risks for metabolic derangements including type 2 diabetes and cardiovascular disease, as well as pro-inflammatory processes. Preterm infants are at higher risk to develop cardiometabolic diseases in later life also, however the desaturase enzyme activity (DA) in this group still inconclusive. In preterm infants, DA may be a rate-limiting step in maintaining PUFAs levels during this critical developmental window. Long chain PUFAs (LCPUFAs) are essential for the structure and function of cell membranes and for growth, with impacts on body composition, immunity, and neurocognition[1]. We defined LCPUFAs based on their molecular structure and are fatty acids with 20 carbons or more and with 2 double bonds or more. Humans are unable to synthesise essential fatty acids de novo and therefore need to be obtained these from the diet, which can enable them to maintain the full component of PUFAs. Desaturase and elongase enzymes play a critical role in the conversion of essential fatty acids and saturated fats to LCPUFAs and MUFAs.

Recent studies in term infants have shown that DA in the first months of life is inversely associated with subsequent body size increase up to 12 months old, but then has positive association from 12 to 24 months, providing a potential tool to predict early life weight gain [2]. Low levels of DA have been associated with insulin resistance, diabetes, and obesity [3, 4]^,^[5]. Preterm infants born before 32 weeks gestation miss out on the transfer of LCPUFAs and are known to be at risk of deficiency of docosahexaenoic acid (DHA) and/or arachidonic acid (ARA) that can result in impaired growth and development, [6, 7] as well as increased risk of cardiovascular disease and metabolic syndrome in later life [8, 9] These deficiencies may not only be caused by DHA availability, DA due to or in combination with precursor deficiency can also limit the availability of DHA and ARA for healthy development in preterm infants [6].

There are three main desaturase enzymes identified in humans: delta 5 desaturases (D5D), delta 6 desaturases (D6D) and delta 9 desaturase (D9D). D5D and D6D are important in LCPUFAs synthesis, while D9D plays a key role in the synthesis of MUFAs [10]. There is limited data on DA in preterm infants, in part due to the challenges of blood sampling in these very small infants. The research team have developed an innovative methodology that allows detailed lipidomic analyses from the small volumes of blood that can be collected onto filter paper as dried blood spots (DBS). The collected DBS allowed us to use them repeatedly because small samples were used to look at changes over time. This is an advantage for a biological study done in babies as the method could optimize the blood volume withdrawn.

This study aimed to explore changes in DA using indirect approach to estimate DA in very preterm (VPT) infants in the first weeks of life and compare with that of healthy term infants. The study hypothesized that desaturase enzyme activity indices are altered in preterm infants compared to term infants in early life and may be a marker of risk or contribute to later alterations in metabolic health.

## Methods

This is comparative analyses of data from two cohorts. Preterm infants were part of a single centre study of preterm lipid metabolism. Infants were prospectively recruited from the neonatal intensive care unit (NICU) from July 2016 - June 2019 at Rosie Hospital, Cambridge University Hospitals NHS Foundation Trust (CUH). Inclusion criteria were gestational age at birth < 32 completed weeks and parental consent with exclusion factors including serious congenital defects, life threatening illness, treatment with antibiotics, necrotizing enterocolitis or serious gastrointestinal abnormalities. The term infants were participants from the Cambridge Baby Growth Study - Breastfeeding (CBGS-BF), enrolled in the study between 2015 and 2019. The cohort excluded antenatal mother with significant comorbidities, and their profile has been described previously [11, 12].

### Sample collection

In the preterm cohort, samples were collected at two time points: (i) within 48 h of establishing full enteral feeds (preterm established enteral feeding, PEF), (ii) at term corrected age (preterm at term, PAT). Term was defined as > 37 weeks postmenstrual age. For the term cohort, samples were taken at birth, two weeks, and six weeks postnatal age. Heel prick blood samples were collected onto untreated filter paper cards (Guthrie cards, Ahlstrom 226; ID Biological Systems, Greenville, South Carolina) as single drops of blood on defined circles (x5). The cards were left to dry naturally for 24 h then stored at -80 °C until batch analyses. Clinical data including antenatal history, clinical course, and feeding history were collected prospectively from the hospital medical records, and from validated parental questionnaires.

### Sample processing

Dried blood spot (DBS) samples were first extracted for determination of lipid species and their relative abundances, using mass spectrometry, as described previously [13]. The lipidomic analysis was done within 2015–2019 for both cohorts, and we did not re-run the lipidomic analysis again for this paper. Instead, we used only their results. Previous studies have shown that lipids in dried blood spot samples are prone to oxidation, but that the effect on inter-individual lipids is limited [13]. To minimise the effect of storage, the samples were stored at -80 degrees until analysis. Product-to-precursor ratio of lipids was measured as a surrogate marker for DA, as reported in previous studies [2, 14, 15]. For this study, D9D was estimated by PC(32:1)/PC(32:0) ratio, whereas PC(38:4)/PC(38:3) and PC(36:4)/PC(36:3) ratios represented D5D enzyme activity. D6D was approximated by the PC(36:3)/(36:2) and TG(52:3)/TG(52:2) ratios. This approach estimates desaturase enzyme activity through abundance ratio analysis. The method compares particular lipids found in the same group, person, and phenotype to provide information on the enzymatic activity related to fatty acids synthesis and modification. This approach has been applied previously in human and mice studies which formulised the usage of these specific PC and TGs as a proxy to estimate DA [16, 2, 15]. These indices were felt to be the most appropriate to use for this study cohort as it requires only very small volumes of blood which is necessary for the preterm babies’ studied. It is also independent of the absolute concentrations of essential fatty acids.

### Analyses

Demographic data was analysed using SPSS version 23.0 for windows 10 (IBM Corp Released 2012.Armonk, NY). Weight data was converted to sex and age-adjusted standard deviation scores (SDS) using the British 1990 growth reference and the WHO 2006 growth standard. The LMS growth was used to calculate standard deviation scores (SDS) in Microsoft Excel [17, 18]. Demographic data is presented as mean standard deviation (SD) and frequencies.

The raw lipid data was initially normalized through log transformation in Metaboanalyst 5.0 and SPSS [19]. Respective lipid values then were calculated into five lipid ratios, as described previously. These lipids ratio which represents D9D, D5D and D6D were checked for normality test. One preterm subject (in PEF group) was identified as an outlier and was therefore removed from further analyses. The data was then tested with T-Test/Mann-Whitney or ANOVA/Kruskal Wallis in SPSS to get further details on mean/median differences (+/- SD) between the cohort.

The trajectories of lipid ratios were explored through Metaboanalyst 5.0. Bonferroni correction for multiple testing was also applied as lipidome analysis consist of large numbers of variables and comparisons. Bonferroni corrected *P* value was used based on dividing the significance threshold of 0.05 by the number of lipids analysed. DA at specific time points and trajectories over time were compared between cohorts by plotting indices with respect to both gestational age and postnatal age. Post hoc analyses were undertaken to explore the effect of extreme prematurity with preterm infants divided further into very preterm and extremely preterm.

## Results

Data was available for a total of 78 preterm infants and 256 term infants. Preterm infants were born at mean (SD) of 28(2.2) weeks gestational age and term infants had a mean (SD) gestational age of 39 (2.2) weeks, with the preterm infants being statistically smaller than the term infants at all study assessments. The majority of all infants in all groups received breast milk. Further demographic details are provided in Table 1.

**Table 1:** Clinical Characteristics

Characteristics	Preterm Cohort		Term Cohort		
	PEF (n = 67)	PAT (n = 38)	Birth (n = 82)	2 weeks post delivery (n = 93)	6 weeks post delivery (n = 81)
Gestational Age at Birth (weeks)	28.00 (2.2)	28.00 (2.3)	39.77 (1.2)	39.77 (1.2)	39.88 (1.1)
Age at assessment	4 (2) weeks	10 (3) Weeks	5 (2) days	2 (1) weeks	6 (0) weeks
Sex	♂: 28 (40%) ♀: 42 (60%)	♂:20 (53%) ♀:18 (47%)	♂:39 (47%) ♀:43 (53%)	♂:39 (42%) ♀:54 (58%)	♂:33 (41%) ♀:48 (59%)
Weight at birth (g)	1117 (407)	1125 (389)	3605 (505)	3657 (487)	3680 (537)
Weight SDS at birth	-0.34 (1.25)	-0.37 (1.31)	0.41 (0.92)	0.51 (0.89)	0.50 (1.08)
Weight at assessment (g)	1456 (422)	2488 (555)	3593 (492)	3981 (612)	4866 (115)
Weight SDS at assessment	-1.81 (2.16)	-1.61 (1.39)	0.046 (0.87)	0.13 (0.99)	0.57 (1.18)
Feeding type	BM 42 (60%) FM 7 (10%) MF 18 (27%)	BM 22 (58%) FM 10 (26%) MF 6 (16%)	BM 71 (87%) FM 4 (5%) MF 6 (7%) NA 1(1%)	BM 81 (87%) FM 2 (2%) MF 10 (11%)	BM 64 (79%) FM 4 (5%) MF 13 (16%)

Abbreviation: PEF –Preterm established on enteral feeding, PAT – Preterm at Term, BM – Breast milk, FM – Formula Milk, MF – Mixed Feeding. Data is given as n (%) or mean (SD) NA – not available

### Desaturase enzyme indices in Preterm Infants

**Fig. 1:**
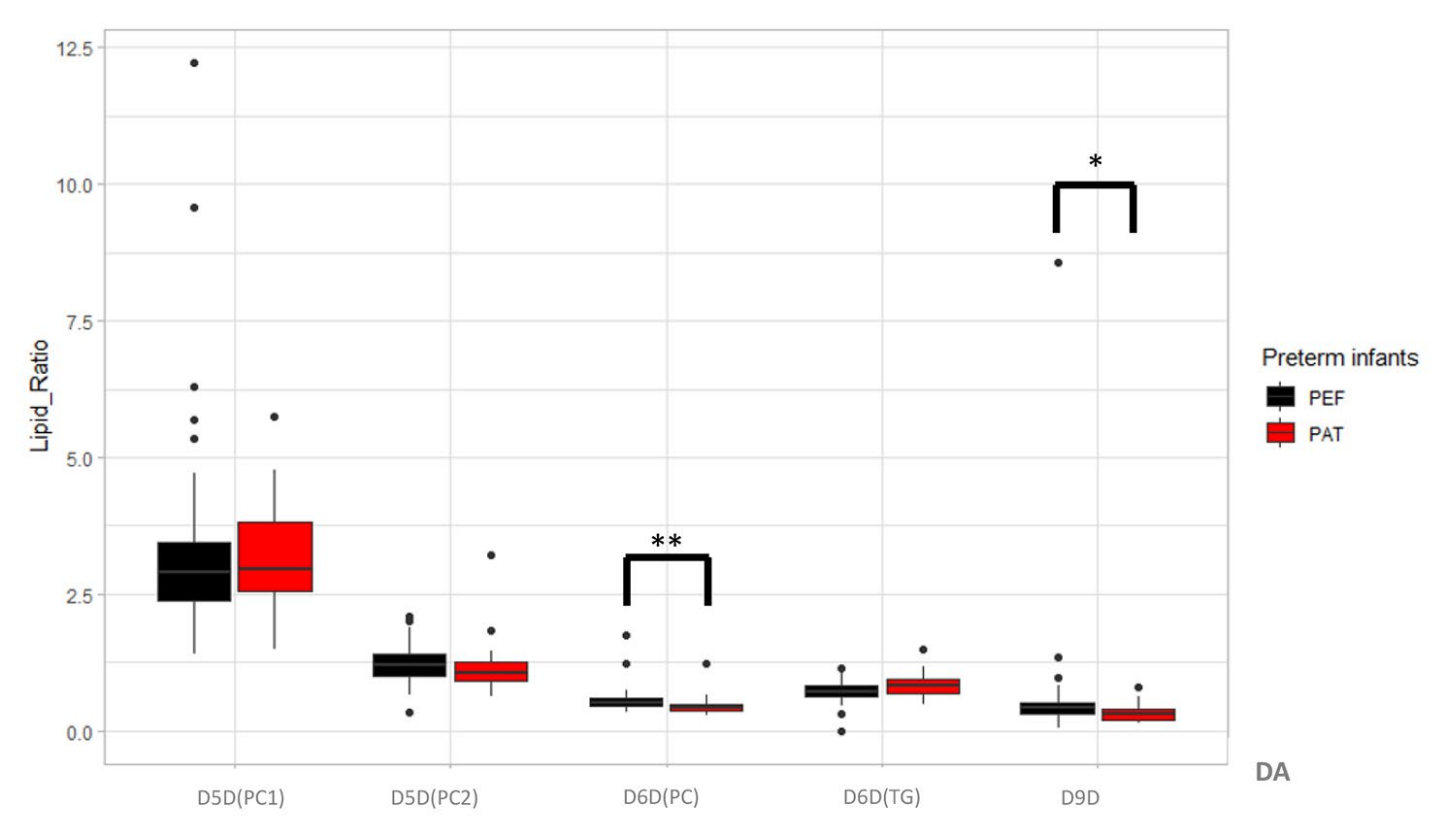
Desaturase Activity in Preterm Infants

As shown in Fig. 1 there were significant differences in specific desaturases indices between the two study time points. D5D (PC2), D6D(PC) and D9D indices levels were lower at term corrected age, compared to when preterm infants were first established on enteral feeds. In contrast D5D (PC1) and D6D(TG) indices levels were higher at term corrected age.

Box plots of the lipid ratios, represent desaturase enzyme activity in preterm infants at two-time points; preterm on establishment of enteral feeding (PEF) and preterm at term corrected age (PAT). The boxes indicate 25th -75th percentile and a median line. Values are in mean (SD). *P* values were obtained by the Mann-Whitney U test. *P < .05, **P < .001.

Abbreviation: PEF-Preterm on established enteral feeding, PAT – preterm at Term, D5D- delta 5 desaturases, (PC): phosphatidylcholine, D5D(PC1): PC(38:4)/PC(38:3), D5D(PC2): PC(36:4)/PC(36:3), D6D- delta 6 desaturases, D6D(PC): PC(36:3)/PC(36:2), TG: triglyceride, D6D(TG): TG(52:3)/TG(52:2), D9D- delta 9 desaturases PC(32:1)/PC(32:0).

### Comparison of DA between preterm and term infants

Table 2 show that PEF infants had similar D9D with term infants at birth, but significantly higher from term infants at 2- and 6-weeks postnatal age (p = .001). At term corrected age (> 37 weeks PMA), D9D was statistically lower than term infants at birth (Table 3). D5D indices was consistently lower in preterm infants at both time points compared to term infants from birth to 6 weeks of age.

**Table 2:** Lipid ratios among preterm infants on establishment of enteral feeds (PEF) and term infants

VARIABLES	PEF	TAB	Z score	*P*	T2W	Z score	*P*	T6W	Z score	*P*
**D9D** **PC(32:1)/PC(32:0)**	0.43 (0.21)	0.43 (0.17)	-0.92	0.36	0.27 (0.11)	-5.88	< 0.001	0.24 (0.13)	-6.59	< 0.001
**D5D** **PC(38:4)/PC(38:3)**	3.08 (1.22)	6.05 (3.87)	-7.11	< 0.001	3.88 (3.36)	-3.21	< 0.05	3.59 (1.05)	-3.98	< 0.001
**D5D** **PC(36:4)/PC(36:3)**	1.23 (0.32)	2.21 (1.06)	-7.64	< 0.001	1.39 (0.34)	-3.09	< 0.05	1.28 (0.26)	-1.37	0.17
**D6D** **PC(36:3)/PC(36:2)**	0.53 (0.12)	0.59 (0.13)	-4.11	< 0.001	0.46 (0.07)	-4.36	< 0.001	0.39 (0.05)	-8.81	< 0.001
**D6D** **TG(52:3)/TG(52:2)**	0.72 (0.16)	0.33 (0.25)	-9.04	< 0.001	0.39 (0.21)	-8.66	< 0.001	0.52 (0.24)	-6.13	< 0.001

The comparison of lipid ratios among PEF and term infants at birth, 2 weeks, and 6 weeks. Lipid ratios were presented as mean (SD), but for statistical significance, the mean ranks among the groups were compared. *P* values were obtained by Kruskal Wallis Test and Post Hoc by Mann Whitney U-Test with Bonferroni adjustment at *P* < .0001, *P* < .05, *P* < .001. Abbreviation: PEF – preterm after establishing enteral feeding, TAB – Term at birth, T2W – Term at 2 weeks, T6W – Term at 6 weeks. D9D- delta 9 desaturases, D5D- delta 5 desaturases 1, D6D- delta 6 desaturases, PC-phosphatidylcholine, TG-triglyceride.

**Table 3:** Lipid ratios among preterm at term (PAT) and term infants

VARIABLES	PAT	TAB	Z score	*P*	T2W	Z score	*P*	T6W	Z score	*P*
**D9D** **PC(32:1)/PC(32:0)**	0.32 (0.16)	0.43 (0.17)	-3.84	< 0.001	0.27 (0.11)	-1.05	0.296	0.24 (0.13)	-2.71	< 0.05
**D5D** **PC(38:4)/PC(38:3)**	3.15 (0.94)	6.05 (3.87)	-5.40	< 0.001	3.88 (3.36)	-1.75	0.081	3.59 (1.05)	-2.22	< 0.05
**D5D** **PC(36:4)/PC(36:3)**	1.13 (0.43)	2.21 (1.06)	-7.08	< 0.001	1.39 (0.34)	-4.74	< 0.001	1.28 (0.26)	-3.80	< 0.001
**D6D** **PC(36:3)/PC(36:2)**	0.46 (0.15)	0.59 (0.13)	-5.86	< 0.001	0.46 (0.07)	-1.93	0.054	0.39 (0.05)	-3.19	< 0.05
**D6D** **TG(52:3)/TG(52:2)**	0.83 (0.20)	0.33 (0.25)	-8.03	< 0.001	0.39 (0.21)	-8.01	< 0.001	0.52 (0.24)	-6.57	< 0.001

Comparison of lipid ratios among PAT and term infants at birth, 2 weeks, and 6 weeks. Lipid ratios were presented as mean (SD), but for statistical significance, the mean ranks among the groups were compared. *P* values were obtained by Kruskal Wallis Test, and Post Hoc by Mann Whitney U-Test with Bonferroni adjustment at *P* < .0001, *P* < .05, *P* < .001. Abbreviation: PAT – preterm at term, TAB – Term at birth, T2W – Term at 2 weeks, T6W – Term at 6 weeks. D9D- delta 9 desaturases, D5D- delta 5 desaturases 1, D6D- delta 6 desaturases, PC-phosphatidylcholine, TG-triglyceride.

D6D in preterm infants was generally higher than term infants at 2 and 6 weeks and especially D6D(TG) indices was significantly higher than term infants, at all-time points. Similarly in PEF infants’, D6D(PC) indices was higher than that of term infants at 2 and 6 weeks (*P* < .001). Although D6D(PC) indices was lower in PAT compared to term infants at birth, it was higher than term infants at 6 weeks (*P* < .03).

**Fig. 2:**
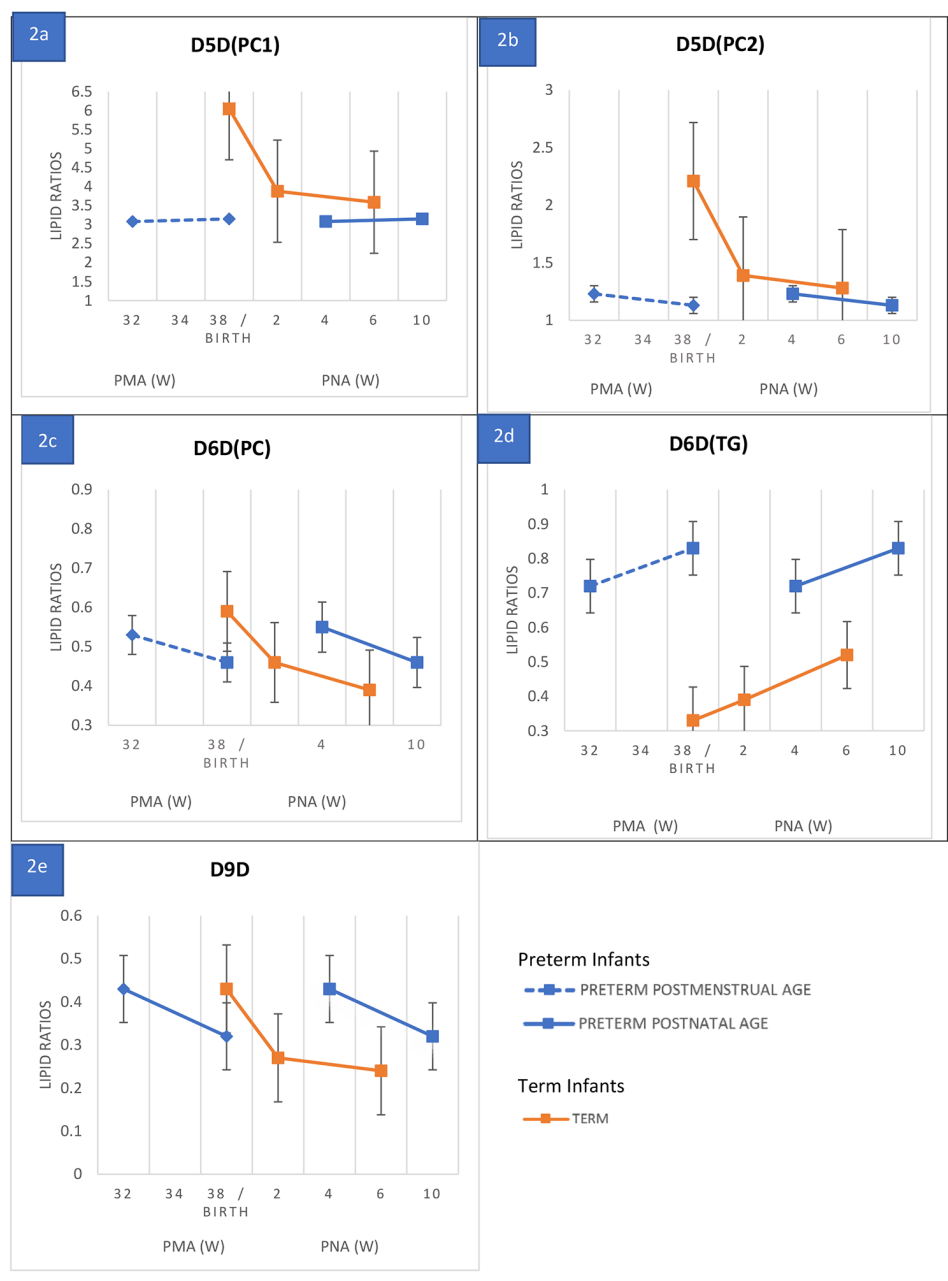
Desaturase Enzyme indices trajectories in preterm and term infants

Desaturase indices in the cohorts over time is shown in Fig. 2, with preterm infant results plotted in duplicate to show levels in relation to postmenstrual (32 and 38 weeks) and postnatal age (4 and 10 weeks). This shows that although there were significant differences in desaturase indices at specific time points, the trajectories of indices over time were similar in the two cohorts. The exception to this was the rapid fall in both D5D indices from birth to 2 weeks postnatal age in the term cohort that was not apparent in the preterm cohort.

Desaturase enzyme indices over time comparing preterm infants with term infants in relation to postmenstrual (32 and 38 gestational age) and postnatal age (4 and 10 weeks). Abbreviations:D5D- delta 5 desaturases, PC - phosphatidylcholine, D5D(PC1) - PC(38:4)/PC(38:3), D5D(PC2)- PC(36:4)/PC(36:3), D6D- delta 6 desaturases, D6D(PC): PC(36:3)/PC(36:2), TG: triglyceride, D6D(TG): TG(52:3)/TG(52:2), D9D- delta 9 desaturases PC(32:1)/PC(32:0), PMA – postmenstrual age, PNA – postnatal age, W – weeks.

## Discussion

These results are the first to show the significant differences in DA indices in a cohort of very preterm infants compared to term controls over the first weeks of life. They also show that although there were significant differences in DA indices at specific time points the trajectory of changes over time were similar in the two cohorts, although offset in terms of absolute levels. As desaturase enzymes play a key role in a number of biochemical pathways including potentially being a rate-limiting step in maintaining LCPUFA levels, altered activity may impact on both short-term metabolism and long-term health. These provisional results would benefit from further studies using isotope tracers to confirm the findings.

Desaturase activity can be affected by many factors including diet with altered gene expression[20, 21]. To avoid the confounding impact of parenteral nutrition preterm infants were only sampled when established on full enteral feeds. In this cohort more preterm infants were receiving formula than term infants, and both volume and composition of breast milk and formula varied between infants. Larger studies would be needed to further investigate the impact of different dietary intakes on desaturase activity.

There is a lack of validated biochemical reference ranges specific for the neonatal population which is a time of significant metabolic adaptation. For preterm infants postmenstrual age may provide a more developmentally appropriate reference compared to postnatal age although nutritional advice for preterm infants is guided both by gestation at birth and postnatal age. This study’s results suggest that changes of desaturase indices in preterm infants are in keeping with the changes seen in term infants from birth to 2-and-6 weeks postnatal age, suggesting reducing trends of MUFAs and LCPUFAs endogenous biosynthesis as infants reach 6–10 weeks postnatal age. Regardless of feeding type, this finding supports that preterm infants have the capacity to convert precursors fatty acids to LCPUFAs and saturated fatty acids to MUFAs as expected for their postnatal age. Further studies would help to determine what changes in activity happen in utero at a comparable gestational age and to determine if the differences in activity persist into childhood.

Preterm infants in this study demonstrated lower D5D and higher D6D indices relative to term cohorts, which may be due to alterations in DA. A series of transition periods, from intrauterine to extrauterine, from parenteral to enteral feeding and apparent growth changes at term corrected age are potentially demanding for metabolic adaptation, but the exact mechanism is indeterminate. It is known that D5D and D6D activity effect the balance of omega-3 and omega-6 synthesis, and that imbalance of omega 3-and-6 can be harmful to human health [9]. In two childhood studies, low D5D and high D6D activity is associated with increased abdominal obesity and correlated to HOMA-IR values [22, 3]. This study did not explore the relationship between DA and either fat mass or insulin resistance but the potential increased risk of altered adiposity in preterm infants in childhood makes this an interesting area of future research as potential biomarker of later metabolism.

These findings are in contrast to a previous study of moderate-to-late preterm infants, which found no difference in D5D and D6D activity compared to term infants. The differences in our findings may be due to the difference in the gestational age of the infants studied. This study selected infants who were very preterm (mean GA 28 weeks) and at most risk of long-term cardiovascular risk compared to the previous study which focused on moderate-to-late preterm infants born between 32 and 36 weeks gestation (mean: 34 +/- 1.7). The infants in this study, who had a mean gestational age at birth of 28 weeks would also have missed the normal in utero placental transfer of DHA and ARA that occurs between 24- and 34-weeks gestation [23]. A further difference was in methodology using total fatty acid ratios as surrogate markers to represent D5D and D6D, rather than specific lipids.

### Strengths and Limitations

The strength of this study was the cohort selections. Very preterm infants were selected as these infants are most at risk from long term metabolic complications of preterm birth as they will have missed the major part of the third trimester, which is the most critical time for LCPUFA transfer. The time-points in this study (PEF and PAT) were important to optimise the confounding factors and useful to observe the consistency of the desaturase indices changes. Another strength of this study was the methodology of lipidomic analysis from DBS paper, which demonstrates the potential for metabolic studies in preterm infants using relatively non-invasive methods and only very small samples of blood.

This study has several limitations including small numbers with fewer preterm at term due to infants being discharged to local units or home prior to reaching term corrected age. Also, the differences in specific postnatal time points in the two cohorts may have led to fatty acid levels measured in the term infants in the first week being raised due to maternal transfer of LCPUFAs compared to the samples taken in the preterm infants at later postnatal ages, with consequences for the estimated DA. However, the decision to measure levels in preterm infants at this time was taken proactively to ensure all infants were fully enterally fed to avoid the potential confounding effect of parenteral nutrition. The methodology used which estimates DA from product/precursor ratios of plasma phosphatidylcholine (PC) and triglyceride (TG) may be affected by differences in dietary intake, but the study was unable to control for this in the clinical setting where preterm and term feeds whether formula or breast milk will vary in composition. In addition, the DA described is based on ratios of specific lipids rather direct measure of enzyme activity, although the ratios reported will be independent of absolute concentrations of essential fatty acids. Given the size of the study the impact of other confounders such as maternal characteristics could not been explored.

## Conclusion

This study shows that in preterm infants desaturase indices change significantly over the first few weeks of life with differences between term and preterm infants at both comparable postmenstrual and postnatal ages. Trajectories of changes suggest these differences may persist over time but require further studies to explore this and the impact on childhood growth and metabolism. This leads to the possibility of identifying potential lipid biomarkers of later cardiometabolic risk and proactive intervention such as dietary modification for early prevention.

### Electronic supplementary material

Below is the link to the electronic supplementary material.

Supplementary Material 1

## Data Availability

The datasets generated during and/or analysed during the current study are available from the corresponding author on reasonable request.

## Abbreviations

ARA: Arachidonic acid
CUH: Cambridge University Hospital
CBGS BF: Cambridge Baby Growth Study Breastfeeding
DA: Desaturase enzyme activity
DBS: Dried blood spot
DHA: Docosahexaenoic acid
D5D: Delta 5 desaturases
D9D: Delta 9 desaturases
LCPUFAs: Long chain polyunsaturated fatty acids
MUFAs: Monounsaturated fatty acids
NICU: Neonatal intensive care unit
PAT: Preterm at term
PEF: Preterm established enteral feeding
PC: Phosphatidylcholine
PMA: Post-menstrual age
PUFAs: Polyunsaturated fatty acids
SDS: Standard deviation score
SD: Standard deviation
TG: Triglyceride
VPT: Very preterm
